# Cutaneous Medicine

**Published:** 1898-01

**Authors:** 


### Book Reviews.

Cutaneous Medicine. A systematic treatise on the diseases of the skin. By Louis A. Duhring, M. D., Professor of Diseases of the Skin in the University of Pennsylvania; author of A Practical Treatise on Diseases of the Skin and Atlas of Skin Diseases. Part II.—classification: Anemia, hyperemia, inflammations. Illustrated. Philadelphia: J. B. Lippincott Company. 1898.

The second part of this important work is in our hands and it is satisfactory to observe that it quite comes up to the expectations raised on seeing the first number. The subjects dealt with are anemia, hyperemia and inflammations.

In order to convey to the mind a correct idea of the arrangement of the diseases of the skin, the author introduces a brief synopsis of the classes as understood by Alibert, Wilson, Hebra, Auspitz, Bronson and Unna to enable the student and physician to conveniently examine and compare these several systems with his own, which, in this work, is a modification and elaboration of the author's old classification, placing special stress upon clinical features and normal anatomy as well as pathological anatomy.

The section dealing with anemia is concise and is discussed under the divisions of general observations—transient anemia, persistent anemia, general pathology and importance in diagnosis. The term hyperemia is used in its strict signification of a superabundance of blood overfilling the blood-vessels of the skin without giving rise to inflammation.

Inflammations are taken up next and gone into, both the superficial and deep-seated varieties, which, of course, include the consideration of the largest well-known list of maladies of which this condition is the basis.

Altogether the features of particular excellence of this work may be summarised as follows : The pathology is clear, up to date, concise and represents a vast amount of research, giving the reader all that is known, free from superfluous or confusing detail. Dr. Duhring again shows his genius in his clean-cut systematology and diagnosis. No dermatological writer can excel him in this particular field. He tersely gives a description so distinct and vivid as to carry to the reader, without question, a picture which becomes real and impressed upon his mind.

The illustrations in this work will be promptly observed as being of exceptional value. It is very difficult to represent by illustration the different features and phases of skin lesions. Much attention has been given to this in the various treatises with but disproportionate success. It can, however, be truthfully stated that those depicted in this work show the lesions and points to be observed, their peculiarities, better than in any other similar publication. No student or practitioner can fail to learn by looking even, nor fail to recognise in the prints the terse description given in the text.

Treatment of skin diseases always permits of latitude and different authorities often hold different views. Duhring gives briefly the various recommendations together with his own and following the tone of the whole work concisely and clear. It may be said that no teacher of dermatology is more successful in his practice, and his formulas, with which his book abounds, are in many instances standard. We can heartily, therefore, for these and many other reasons, recommend the work in the most unreserved way. Were the profession under no previous obligations to Dr. Duhring for his labors, they would be now. E. W.

